# Seroprevalence of Cytomegalovirus and Associated Factors Among Preconception Women: A Cross-Sectional Nationwide Study in China

**DOI:** 10.3389/fpubh.2021.631411

**Published:** 2021-08-25

**Authors:** Qiongjie Zhou, Qiaomei Wang, Haiping Shen, Yiping Zhang, Shikun Zhang, Xiaotian Li, Ganesh Acharya

**Affiliations:** ^1^Obstetrics and Gynecology Hospital of Fudan University, Shanghai, China; ^2^Women's Health and Perinatology Research Group, Department of Clinical Medicine, UiT - The Arctic University of Norway, Tromsø, Norway; ^3^Department of Maternal and Child Health, National Health and Family Planning Commission of the People's Republic of China, Beijing, China; ^4^Shanghai Key Laboratory of Female Reproductive Endocrine-Related Diseases, Shanghai, China; ^5^Department of Clinical Science, Intervention and Technology (CLINTEC), Division of Obstetrics and Gynecology, Karolinska Institute, Stockholm, Sweden

**Keywords:** cytomegalovirus infection, pregnancy, preconception care, China, congenital infection

## Abstract

**Background:** Cytomegalovirus seroconversion during pregnancy is common and has a substantial risk of congenital infection with longterm sequale. Screening during pregnancy or vaccination have not been shown to be effective for eliminating congenital infections. Preconception screening policy has not been evaluated adequately in a large scale. This nationwide study aimed to investigate epidemiological features of cytomegalovirus seropositivity and its geographic variation among Chinese women planning a pregnancy to gather epidemiological evidence as an essential for developing novel prevention strategies.

**Method:** This cross-sectional sero-epidemiological survey enrolled women intending to become pregnant within 6 months in mainland China during 2010–2012. The primary outcomes in this study were cytomegalovirus Immunoglobulin G and M seropositivity. Secondary outcomes were the associations between Immunoglobulin G and Immunoglobulin M, with socio-demographic characteristics, including age, occupation, education level, place of residence, and ethnicity. The overall seropositivity and regional disparity was analyzed on the individual and regional level, respectively.

**Results:** This study included data from 1,564,649 women from 31 provinces in mainland China. Among participants, 38.6% (*n* = 603,511) were cytomegalovirus immunoglobulin G+, 0.4% (*n* = 6,747) were immunoglobulin M+, and 0.2% (*n* = 2,879) were immunoglobulin M+ and immunoglobulin G+. On individual level, participant's age, ethnicity, and residing region were significantly associated with IgG+, IgM+, and IgM+IgG+ (*P* < 0.001), while occupation, education level, and place of residence were not statistically significant (*P* > 0.05). On regional level, cytomegalovirus immunoglobulin G and immunoglobulin M seropositivity was highest in the eastern region (49.5 and 0.5%, respectively), and lowest in the western region (26.9 and 0.4%, respectively). This geographic variation was also noted at the provincial level, characterized by higher provincial immunoglobulin M+ and immunoglobulin G+ rates associated with higher immunoglobulin G seropositivity. In the subgroup analysis of immunoglobulin G seropositivity, areas of higher immunoglobulin G positivity had a higher rate of immunoglobulin M+, indicating an expected increased risk of reinfection and primary infection.

**Conclusions:** A substantial proportion of women (>60%) were susceptible to cytomegalovirus in preconception period in China, and immunoglobulin G seropositivity was seen at a low-medium level with substantial geographic variation. Integration of cytomegalovirus antibody testing in preconception screening program based on regional immunoglobulin G seropositivity, should be considered to promote strategies directed toward preventing sero-conversion during pregnancy to reduce the risk of this congenital infection.

## Background

Maternal-fetal transmission of cytomegalovirus (CMV) is one of the most common causes of congenital infections, and prevention has proven difficult (1–3). It is estimated that, in developed countries, 0.64% of infants are born with congenital CMV infection (presence of the virus in their urine or saliva within 3 weeks of birth) (4). In the United States alone, ~970,000 women of childbearing age experience a primary CMV infection each year (5).

Some studies from China have reported high prevalence of seropositivity (>90%) among pregnant women with a maternal seroprevalence of 92–99% (6–8), but national data are lacking. Furthermore, prevalence among women in preconception period nationally and rate of seroconversion during pregnancy is not known. There is no approved vaccine for CMV although it could be an option for eliminating maternal-fetal transmission (6).

Screening during pregnancy has been suggested by some and targeted screening is practiced in few countries (9), but universal antenatal screening is not recommended (10, 11) due to the lack of effective treatment and difficulty in predicting sequalae. Therefore, insight into potential alternative screening strategies to detect and treat CMV infection before pregnancy and take targeted hygienic measures to prevent infection during pregnancy in susceptible women is needed. As primary infection in the first trimester is associated with highest risk of transplacental transmission, preventive measures should be ideally started before conception (12).

Approximately 36.5% of congenital CMV infections occur within the first 3 months of pregnancy and are secondary to maternal infection (13). Severe fetal abnormalities are linked to congenital CMV infection during the first trimester (13). Sequelae can be serious and include a substantial risk of perinatal mortality, long-term neurodevelopmental disorders, and other severe adverse effects such as sensorineural hearing loss and cognitive impairment (14).

Preconception CMV screening could provide a window of opportunity to avoid maternal-fetal transmission that is not available once a woman becomes pregnant. It has been suggested that women should consider delaying conception for at least 6 months after primary infection to prevent a possible congenital infection. In addition, confirming maternal immunity provides reassurance as the presence of maternal CMV IgG antibodies significantly decreases the risk of fetal infection (15). Whereas, women that are Immunoglobulin (Ig) G negative (do not carry CMV antibodies) are at risk of primary infection and should be advised to take adequate precautions, such as avoiding contact with body fluids from infected persons, during pregnancy.

Population-based preconception screening for CMV infection could potentially reduce the risk of maternal-fetal CMV transmission by targeted preventive measures. However, information on sero-prevalence is essential before such program could be implemented and run cost-effectively.

The objective of this study was to investigate epidemiological features of CMV seropositivity by province among Chinese women planning a pregnancy, and also to identify factors associated with seropositivity.

## Methods

### Data Sources

This study utilized data from the National Preconception Health Care Project (NPHCP). The project was conducted from January 2010 to December 2012 in 220 counties, located in 31 provinces and province level municipalities, in mainland China (16). The National Health and Family Planning Commission of the People's Republic of China (NHFPC) launched the NPHCP in 2010, providing free preconception care to married couples planning a pregnancy in rural areas. Specifically, the project provided free preconception care to women intending to get pregnant within 6 months. In 2013, the program was expanded to all rural areas in mainland China. This study extracted project data from January 2010 through December 2012. The Institutional Review Board of the Chinese Association of Maternal and Child Health Studies approved the project (IRB201001), and written informed consent was obtained from each participant before enrollment.

### Participants

Women planning to conceive were encouraged to participate in the NFPHEP where they resided. The services were provided by local family planning service agencies and maternal- and child-care service centers, which included health examination, health education, health promotion, medical advice and referral to a physician or transfer of care to another healthcare facility as required. Participants were required to be between the ages of 20 and 49 years to participate. Other inclusion criteria included enrollment in the National Preconception Care Project between 2010 and 2012. Women who failed to complete the preconception health examination, did not receive CMV IgG serology testing, or did not provide province information were excluded from analysis.

### Data Collection

During an initial examination, trained health care personnel conducted face-to-face interviews and medical examinations of couples who were identified as those planning pregnancy within 6 months. Data on socio-demographic characteristics including age, place of residence, education level, and occupation that were collected using a standardized questionnaire were extracted for analyses. The participants were grouped according to their residence in one of the 31 provinces and categorized into three regional groups according to residential address. The regional groups were the eastern region (including Beijing, Fujian, Guangdong, Jiangsu, Liaoning, Shandong, Shanghai, Tianjin, and Zhejiang), the central region (including Anhui, Hainan, Hebei, Heilongjiang, Henan, Hubei, Hunan, Jiangxi, Jilin, and Shanxi), and the western region (including Chongqing, Gansu, Guangxi, Guizhou, Inner Mongolia, Ningxia, Qinghai, Shaanxi, Sichuan, Tibet, Yunnan, and Xinjiang).

Based on the guidelines of NHFPC, community healthcare staff inquired about pregnancy intentions and provided participants with preconception healthcare. Health education, medical advice and referral to a physician or transfer to appropriate healthcare facility was provided as required. All survey information and medical examination data were uploaded, transferred remotely, and stored in the NFPHEP medical service information system, which was supported and operated by the National Research Institute for Family Planning. Detailed design, organization, implementation, and quality control of the project have been described elsewhere (16–19). Results of CMV serology tests and socio-demographic information were extracted for analyses from the NFPHEP medical service information system.

### CMV Serology Test

During the preconception examination, 5 mL of venous blood was collected from each participant and samples were stored at −30°C. All serum specimens were analyzed for CMV IgG and IgM using commercially available enzyme immuno-assay kits for the detection of IgG and IgM antibodies according to the manufacturers' instructions in local laboratories (8). Reagent kits approved by the China Food and Drug Administration were selected for use at the discretion of the local laboratories. A detailed quality control system was used to ensure that the diagnostic capability of the test kits was comparable. A series of documents were also published describing the standards of sampling, clinical sample storage and transportation procedures, quality control protocols, and standards of clinical testing (17).

The CMV infection and transmission depends on individual susceptibility as well as other population-based factors affecting transmission and immunity. The CMV serology is not only related to primary infection, but recurrent infection as well. The CMV serology test results in our study were interpreted as follows: (1) women with positive IgG (IgG+) and negative IgM (IgM-) were considered to have had previous infection/immunization and to be at low risk; (2) women with negative IgG (IgG-) and IgM- were susceptible to infection and advised to obtain health education before conception; and (3) women with positive IgM (IgM+) and IgG+ or IgG- were referred to a specialist for further diagnostic examination and treatment.

### Statistical Analysis

We analyzed the data of women who had completed the preconception health program in the NPHCP database. We extracted the socio-demographic variables of the participants that were available, including maternal age, education level, occupation, residence, and ethnicity. Descriptive data are presented as mean (SD), median (range) or *n* (%) as appropriate. The statistical analysis was conducted on both individual and regional level. On individual level, we analyzed the factors that might be related with CMV serology. On regional level in provincial units, geographic disparity and association between IgG and IgM were analyzed. A multivariate correlation analysis was used to determine the association between CMV serology (IgG and IgM) and geographic variables adjusted by maternal age, education level, and occupation. A *P*-value <0.05 was considered statistically significant. Statistical analyses were performed using SPSS version 26.0 (IBM Corp., NY, USA).

## Results

### CMV Seroprevalence

A total of 1,564,649 (73.0%) women were included in the final analysis. The percentage of participants that tested CMV IgG+ was relatively low (38.6%, *n* = 603,511). The percentage of IgM+ participants was 0.4% (*n* = 6,747). Among all participants, 0.2% (*n* = 2,879) tested positive for both IgM and IgG, and 0.2% (*n* = 3,868) tested positive for IgM and negative for IgG.

### Association With Socio-Demographic Characteristics of Participants

On individual level, demographic characteristics in relation to CMV serology test results are described in Table 1. The seropositivity status, i.e., IgG+, IgM+, or IgM+IgG+, was significantly different among women with different age, ethnicity, and residing region (*P* < 0.001), while the differences in seropositivity according to occupation, education level, and place of residence were not statistically significant (*P* > 0.05).

**Table 1:** Demographic and regional characteristics of cytomegalovirus serology.

	**Total**	**IgG+**	**IgM+**	**IgM+ IgG+**	**IgM+ IgG-**	***P*-value^**1**^**	***P*-value^**2**^**	***P*-value^**3**^**	***P*-value^**4**^**
Age (years)						<0.001	<0.001	<0.001	<0.001
21–29	35,9881	93,257 (25.9)	1,678 (0.5)	640 (0.2)	1,038 (0.3)				
30–39	86,805	24,865 (28.6)	554 (0.6)	253 (0.3)	301 (0.3)				
40–49	10,506	2,559 (24.4)	78 (0.7)	33 (0.3)	45 (0.4)				
Occupation						0.082	<0.001	0.078	<0.001
Farmers	124,4597	47,4264 (38.0)	5,293 (0.4)	2,250 (0.2)	3,043 (0.2)				
Workers	12,0149	43,856 (36.5)	545 (0.5)	215 (0.2)	330 (0.3)				
Employers	173,074	74,459 (43.0)	798 (0.5)	353 (0.2)	445 (0.3)				
Housewives	26,829	10,935 (40.8)	111 (0.4)	59 (0.2)	52 (0.2)				
Education						0.195	<0.001	0.552	<0.001
Illiteracy or primary school	119,927	43,303 (36.1)	577 (0.5)	273 (0.2)	304 (0.3)				
Junior high school	103,5848	39,1132 (37.8)	4,328 (0.4)	1,806 (0.2)	2,522 (0.2)				
Senior high school	262,823	99,873 (38.0)	1,150 (0.4)	512 (0.2)	638 (0.2)				
College or higher	146,051	69,203 (47.4)	692 (0.5)	288 (0.2)	404 (0.3)				
Residence						0.052	<0.001	0.334	<0.001
Rural	148,2568	568,375 (38.3)	6,428 (0.4)	2,718 (0.2)	3,710 (0.3)				
Urban	82,081	35,136 (42.8)	319 (0.4)	161 (0.2)	158 (0.2)				
Ethnicity						<0.001	<0.001	<0.001	<0.001
Han	145,2147	31,506 (38.9)	6,192 (0.4)	2,547 (0.2)	3,645 (0.3)				
Non-Han	31,263	13,782 (44.1)	75 (0.2)	50 (0.2)	25 (0.1)				
Region						<0.001	<0.001	<0.001	<0.001
East	274,101	135,758 (49.5)	1,427 (0.5)	669 (0.2)	758 (0.3)				
Middle	796,820	335,1666 (42.1)	3,431 (0.4)	1,481 (0.2)	1,950 (0.2)				
West	493,728	132,587 (26.9)	1,889 (0.4)	729 (0.1)	1,160 (0.2)				
IgG positivity, %						<0.001	<0.001	<0.001	<0.001
≥80	26,646	23,161 (86.9)	96 (0.4)	85 (0.3)	11 (0.0)				
40–79	604,178	329,871 (54.6)	3,057 (0.5)	1,400 (0.2)	1,657 (0.3)				
s ≤ 40	933,825	250,479 (26.8)	3,594 (0.4)	1,394 (0.2)	2,200 (0.2)				
Total	1564,649	603,511 (38.6)	6,747 (0.4)	2,879 (0.2)	3,868 (0.2)				

*Data were presented as number (percentage)*.

*P-value^1^: for IgG+*.

*P-value^2^: for IgM+*.

*P-value^3^: for IgM+ and IgG+*.

*P-value^4^: for IgM+ and IgG-*.

*All participants were grouped into 31 provinces and divided into three regions: eastern region (including Beijing, Fujian, Guangdong, Jiangsu, Liaoning, Shandong, Shanghai, Tianjin, and Zhejiang), central region (including Anhui, Hainan, Hebei, Heilongjiang, Henan, Hubei, Hunan, Jiangxi, Jilin, and Shanxi), and western region (including Chongqing, Gansu, Guangxi, Guizhou, Inner Mongolia, Ningxia, Qinghai, Shaanxi, Sichuan, Tibet, Yunnan, and Xinjiang) according to their residential address. P-value for the association between CMV serology and geographic variables were adjusted by maternal age, education level, and occupation by multivariate correlation analysis*.

### Association With Geographic Factors

**Figure 1:**
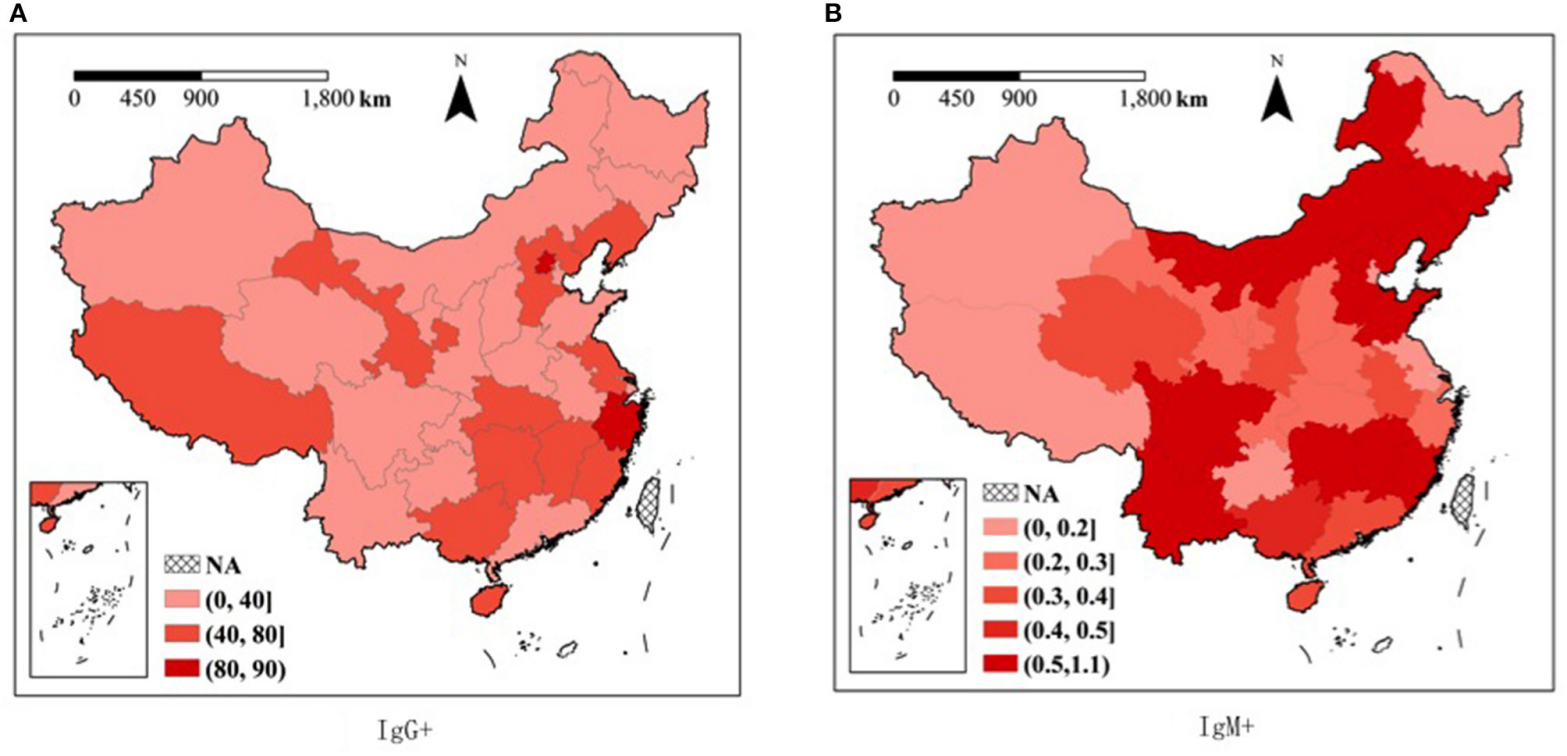
Cytomegalovirus serology by provinces in China. **(A)** CMV IgG; **(B)** CMV IgM.

On regional level, the geographic factors significantly contributed to the results of CMV serology. Examination of the geographic variation of serology test results showed CMV IgG seropositivity ranging from highest in the eastern region (49.5%) to lowest in the western region (26.9%). The CMV serology test results (IgG+, IgM+, IgM+IgG+, and IgM+IgG-) were significantly associated with the region of residence in this subgroup analysis (*P* < 0.05) (Table 1). In addition, a substantial geographical variation was also seen at the provincial level. Differences in CMV IgG seropositivity ranged from 4.8% in the Ningxia Province to 88.8% in Beijing. The highest IgM+IgG+ rate was observed in Beijing (0.6%), and the lowest rate was observed in Tianjing (0.0%), Tibet (0.0%), and Ningxia (0.0%) (Figure 1 and Supplementary Table 1).

**Figure 2:**
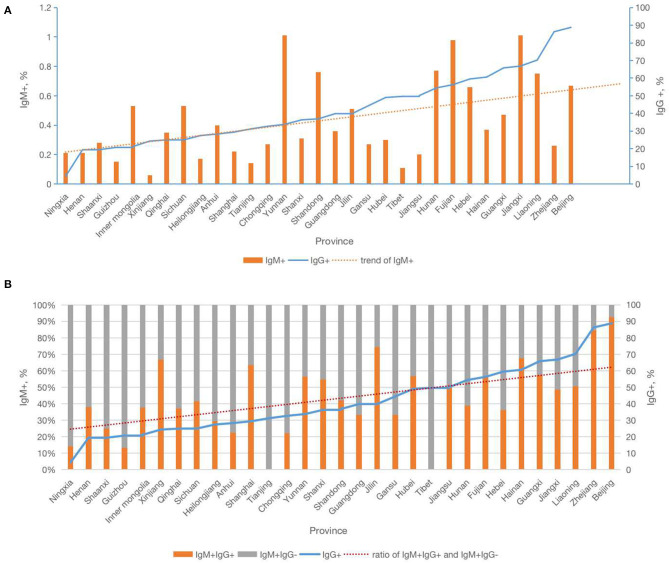
Relationship of Cytomegalovirus IgG and IgM serology by provinces in China. **(A)** The relationship of Cytomegalovirus IgM+ and trend of IgM+ with IgG+ by provinces in China. **(B)** The relationship of Cytomegalovirus IgM+IgG+ with trend of IgG+ and ratio of IgM+IgG+ and IgM+IgG− by provinces in China.

Analysis of the correlation of CMV serology test results demonstrated that provincial CMV IgM+IgG+ rates were significantly correlated with provincial CMV IgG+ rates. In the subgroup analysis of varying IgG positive groups, higher IgG+ areas also had higher rates of IgM+IgG+. The highest rates IgM+IgG+ were observed in areas where IgG+ was >80%, and the lowest rates of IgM+IgG+ were observed in areas where IgG+ was <40% (Table 1). At the provincial level, the analysis indicated that the rate of IgM+IgG+ and the ratio of IgM+IgG+ to IgM+IgG- increased as the rate of IgG seropositivity increased; however, the IgM+IgG- rate was not significantly correlated with the rate of IgG seropositivity (Figure 2 and Supplementary Figure 1).

## Discussion

This nationwide study of integrated preconception screening demonstrated the epidemiological features of CMV seropositivity and its geographic variation among Chinese women planning a pregnancy. We found that overall CMV IgG seropositivity and IgM+IgG+ rates in women in the preconception period in China were 38.6 and 0.2%, respectively, and were characterized by a substantial geographic variation. Furthermore, our analysis indicated that preconception CMV serology screening is useful in identifying women with possible infection before pregnancy that would benefit from further IgG avidity testing, as women with low IgG avidity, suggesting recent infection, could benefit from appropriate counseling regarding pregnancy planning to prevent maternal-fetal CMV transmission.

**Figure 3:**
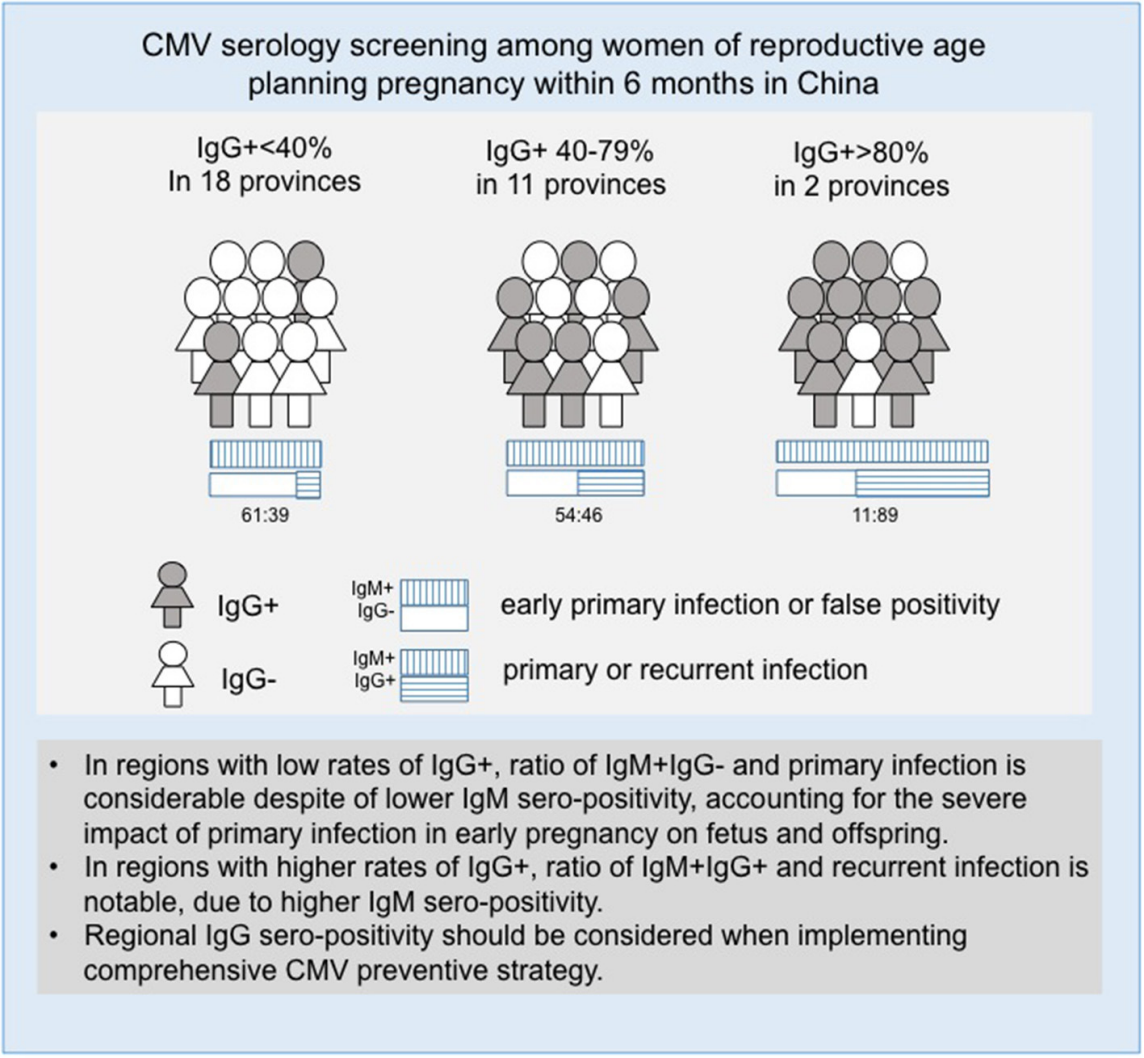
Diagram of CMV serology before pregnancy in China.

Preconception screening provides a unique opportunity to identify infected and treat women before pregnancy. Screening in antenatal care is useful for identifying primary infection, and first trimester screening may contribute to preventing congenital infection related handicap as it allows the susceptible women to focus on preventive measures to avoid infection especially during periconception period and first trimester of pregnancy when the risk of maternal-fetal CMV transmission is highest (12). The regional serological variations and sero-prevalence associations that were found in our study suggest that a targeted preconception testing strategy based on regional CMV IgG serology prevalence could also help to decrease the risk of congenital CMV infection nationwide (Figure 3).

To our knowledge, this is the largest study describing the sero-epidemiology of CMV in women in the preconception period worldwide. The main strength of this study is that it included participants from all 31 provinces in mainland China (Supplementary Table 1). The study illustrates a strategy how congenital CMV infection could be prevented if CMV infection is diagnosed and treated before conception. The design of the preconception screening program analyzed in this study has the potential to identify CMV infection before conception, therefore, reducing maternal-fetal CMV transmission.

The kits used for CMV serology testing were not the same across all the laboratories and provinces. However, the detection limits of CMV IgG and IgM seropositivity as a categorical variable (sero-positive or sero-negative) were comparable across laboratories under a well-implemented quality control program. Although the reagents, kits, quantitative levels of antibodies, and cut-off values varied, only kits that were approved by the China Food and Drug Administration were used in the project. Moreover, several quality control mechanisms were in place to ensure that the appropriate procedures were followed for sampling, transporting, and storing clinical samples. In addition, laboratory testing and reporting were conducted in accordance with national standards.

We found that 0.2% of women overall had a recent CMV infection, or a reactivation or reinfection before conception, with the highest and the lowest in eastern and western regions, respectively. The significant geographic disparity of the prevalence of CMV seropositivity could, in part, be due to cultural, social and behavioral values, associated with personal hygiene, unbalanced economic development, population density, and disparity in access to health care. Implementation of targeted health education and health care measures in preconception period based on the analysis of these factors could be useful in reducing congenital infections.

Overall, CMV IgG+ rates among Chinese women before conception were found to be relatively low (38.2%) compared to the seropositivity rates reported during pregnancy (6–8). For example, the rate of IgG seropositivity in the preconception period in Jiangsu province was 50.1% in our study. This is substantially lower than the IgG seropositivity reported among pregnant women (98.7%) in the same province (7). In our study, we did not find an increased prevalence of seropositivity with increasing maternal age. This difference could possibly be explained by differences in the populations studied, i.e., women planning pregnancy vs. pregnant women. Whether the different time periods when the studies were performed could have influenced the results also needs to be considered.

Serological CMV screening during preconception period also presents potential benefits for possible vaccination of susceptible women avoiding the risk during pregnancy. There is growing evidence that CMV vaccination could protect adults and children from infection, and largely known vaccination targets and defined paths for licensure have been established (20–22). In several clinical trials, vaccination is considered to be indicated for seronegative women when addressing primary CMV infection. However, recent studies have shown that neonates with congenital CMV infection are born to women with non-primary infection (23–26). The exact frequency of CMV non-primary infection and intrauterine transmission among seroimmune pregnant women is still unclear (27). Further investigation is required to understand of the source of virus which leads to non-primary maternal infection to reduce the incidence of congenital CMV among pregnant women in regions with high CMV seroprevalence (28). In addition, whether using valacyclovir during pregnancy for prevention and treatment of congenital CMV infection is beneficial still remains to be confirmed (29). In an observational study, hyperimmunoglobulin treatment at a biweekly dose of 200 IU/kg seemed to be potentially useful among women with a recent primary infection in the first trimester or during the periconception period (30). Thus, offering effective vaccination to susceptible women before conception may also be possible in the near future.

The association between IgM and IgG serology indicated that participants in areas with higher rates of IgG seropositivity are more likely to be seropositive due to secondary infection, while those in areas of lower IgG seropositivity are more likely to be sero-positive due to primary infection. This finding suggests that the development of a preventive strategy should be targeted based on the rate of IgG seropositivity in a geographic area. The regional differences in serology test results found in this study provide baseline data for future analysis of infection characteristics.

Our study does have limitations. First, the pregnancy outcomes in the screened population and prevalence of congenital CMV among the neonates were not analyzed in this study. Further studies are needed to evaluate the impact of preconception screening and its cost-effectiveness. Second, the proportion of women with different ethnicity varied by provinces and could possibly represent selection bias. However, as this nationwide study included all 31 provinces and 50 of 56 races in mainland China, we believe our findings are generalizable to the whole country. Third, although several socio-demographic factors were considered in this study, association of CMV seropositivity with other confounders, such as high-risk occupations, such as child-carers and medical staff, were not evaluated.

## Conclusion

A substantial proportion of women (>60%) were suspectable to cytomegalovirus in preconception period in China, and immunoglobulin G seropositivity was seen at a low-medium level with substantial geographic variation. Integration of cytomegalovirus antibody testing in preconception screening program based on regional immunoglobulin G seropositivity, should be considered to promote strategies directed toward preventing seroconversion during pregnancy to reduce the risk congenital cytomegalovirus infection.

## Data Availability Statement

Dataset analyzed in this study was based on the national database and public access to the database is closed. Zhang Shikun gave the administrative permission to access the database on behalf of National Health and Family Planning Commission of the People's Republic of China (NHFPC).

## Ethics Statement

The Institutional Review Board of the Chinese Association of Maternal and Child Health Studies approved the project (IRB201001), and written informed consent was obtained from each participant before enrollment.

## Author Contributions

QZ and XL carried out the statistical analysis and drafted the manuscript. GA interpreted data and drafted the manuscript. SZ, QW, HS, and XL participated in the design of the study and coordination. All authors contributed to the article and approved the submitted version.

## Author Disclaimer

The views expressed in this report are those of the authors and do not necessarily reflect the official policy or position of the Department of Maternal and Child Health of National Health and Family Planning Commission (NHFPC) in China.

## Conflict of Interest

The authors declare that the research was conducted in the absence of any commercial or financial relationships that could be construed as a potential conflict of interest.

## Publisher's Note

All claims expressed in this article are solely those of the authors and do not necessarily represent those of their affiliated organizations, or those of the publisher, the editors and the reviewers. Any product that may be evaluated in this article, or claim that may be made by its manufacturer, is not guaranteed or endorsed by the publisher.

## Supplementary Material

Supplementary Figure 1 **(A)** The relationship of Cytomegalovirus IgM+IgG+ with IgG+ and trend of IgM+IgG+ by provinces in China. **(B)** The relationship of Cytomegalovirus IgM+IgG− with IgG+ and trend of IgM+IgG− by provinces in China.
