# Supplementary material for: Seroprevalence of Cytomegalovirus and Associated Factors Among Preconception Women: A Cross-Sectional Nationwide Study in China
Source: Front Public Health. 2021 Aug 25;9:631411. doi: 10.3389/fpubh.2021.631411 (PMC8425481; doi:10.3389/fpubh.2021.631411)
Supplement: Supplementary file 1 [file Table_1.DOCX]

Supplemental Table 1. Provincial cytomegalovirus serology in China.

|  | Total number | IgG+ |  | IgM+ |  | IgM+ and IgG+ |  | IgM+ and IgG- |  |
| --- | --- | --- | --- | --- | --- | --- | --- | --- | --- |
|  |  | Number | ％ | Number | ％ | Number | ％ | Number | ％ |
| Anhui | 104389 | 29554 | 28.3% | 421 | 0.4% | 97 | 0.1% | 324 | 0.3% |
| Beijing | 6591 | 5853 | 88.8% | 44 | 0.7% | 41 | 0.6% | 3 | 0.1% |
| Fujian | 14962 | 8426 | 56.3% | 146 | 1.0% | 82 | 0.6% | 64 | 0.4% |
| Gansu | 45831 | 20380 | 44.5% | 126 | 0.3% | 40 | 0.1% | 86 | 0.2% |
| Guangdong | 71829 | 28627 | 39.9% | 259 | 0.4% | 85 | 0.1% | 174 | 0.2% |
| Guangxi | 12011 | 7904 | 65.8% | 56 | 0.5% | 32 | 0.3% | 24 | 0.2% |
| Guizhou | 79738 | 16518 | 20.7% | 121 | 0.2% | 13 | 0.0% | 108 | 0.1% |
| Hainan | 16408 | 9947 | 60.6% | 61 | 0.4% | 41 | 0.3% | 20 | 0.1% |
| Hebei | 128229 | 76336 | 59.5% | 847 | 0.7% | 310 | 0.2% | 537 | 0.4% |
| Henan | 166475 | 32272 | 19.4% | 347 | 0.2% | 133 | 0.1% | 214 | 0.1% |
| Heilongjiang | 15445 | 4235 | 27.4% | 26 | 0.2% | 7 | 0.1% | 214 | 0.1% |
| Hubei | 206548 | 101298 | 49.0% | 615 | 0.3% | 356 | 0.2% | 259 | 0.1% |
| Hunan | 69724 | 37958 | 54.4% | 539 | 0.8% | 210 | 0.3% | 329 | 0.5% |
| Jilin | 34638 | 13803 | 39.9% | 178 | 0.5% | 132 | 0.4% | 46 | 0.1% |
| Jiangsu | 41312 | 20708 | 50.1% | 82 | 0.2% | 43 | 0.1% | 39 | 0.1% |
| Jiangxi | 32104 | 21459 | 66.8% | 325 | 1.0% | 156 | 0.5% | 169 | 0.5% |
| Liaoning | 34236 | 24058 | 70.3% | 257 | 0.8% | 130 | 0.4% | 127 | 0.4% |
| Inner mongolia | 28997 | 6160 | 21.2% | 155 | 0.5% | 58 | 0.2% | 97 | 0.3% |
| Ningxia | 28766 | 1256 | 4.4% | 59 | 0.2% | 8 | 0.0% | 51 | 0.2% |
| Qinghai | 15937 | 3979 | 25.0% | 56 | 0.4% | 20 | 0.1% | 36 | 0.2% |
| Shandong | 75026 | 27694 | 36.9% | 570 | 0.8% | 239 | 0.3% | 331 | 0.4% |
| Shanxi | 22860 | 8304 | 36.3% | 72 | 0.3% | 39 | 0.2% | 33 | 0.1% |
| Shaanxi | 77215 | 15000 | 19.4% | 219 | 0.3% | 53 | 0.1% | 166 | 0.2% |
| Shanghai | 3656 | 1075 | 29.4% | 8 | 0.2% | 5 | 0.1% | 3 | 0.1% |
| Sichuan | 68004 | 16996 | 25.0% | 360 | 0.5% | 149 | 0.2% | 211 | 0.3% |
| Tianjing | 6434 | 2009 | 31.2% | 9 | 0.1% | 0 | 0.0% | 9 | 0.1% |
| Tibet | 2813 | 1397 | 49.7% | 3 | 0.1% | 0 | 0.0% | 3 | 0.1% |
| Xinjiang | 18550 | 4515 | 24.3% | 12 | 0.1% | 8 | 0.0% | 4 | 0.0% |
| Yunnan | 54708 | 18481 | 33.8% | 555 | 1.0% | 311 | 0.6% | 244 | 0.5% |
| Zhejiang | 20055 | 17308 | 86.3% | 52 | 0.3% | 44 | 0.2% | 8 | 0.0% |
| Chongqing | 61158 | 20001 | 32.7% | 167 | 0.3% | 37 | 0.1% | 130 | 0.2% |
| Total | 1564649 | 603511 | 38.6% | 6747 | 0.4% | 2879 | 0.2% | 3868 | 0.3% |
