# Supplementary figures and images for: Seroprevalence of Cytomegalovirus and Associated Factors Among Preconception Women: A Cross-Sectional Nationwide Study in China
Source: Front Public Health. 2021 Aug 25;9:631411. doi: 10.3389/fpubh.2021.631411 (PMC8425481; doi:10.3389/fpubh.2021.631411)

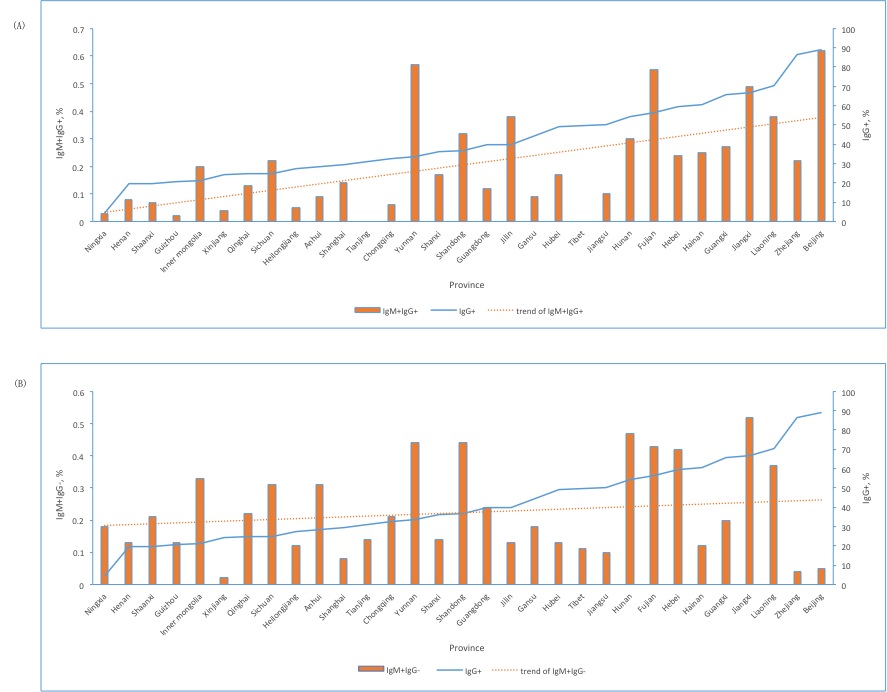

Supplement: Supplementary Figure 1 — (A) The relationship of Cytomegalovirus IgM+IgG+ with IgG+ and trend of IgM+IgG+ by provinces in China. (B) The relationship of Cytomegalovirus IgM+IgG− with IgG+ and trend of IgM+IgG− by provinces in China. [file Image_1.jpg]
